# Retraction: The two-component system CpxRA negatively regulates the locus of enterocyte effacement of enterohemorrhagic *Escherichia coli* involving σ^32^ and Lon protease

**DOI:** 10.3389/fcimb.2025.1639671

**Published:** 2025-06-12

**Authors:** Frontiers Editorial Office

The journal retracts the 05 February 2016 article cited above.

Following publication, concerns were raised regarding the integrity of the images in the published figures. Image duplication concerns were identified within Figure 1 and Figure 3. The authors failed to provide a satisfactory explanation during the investigation, which was conducted in accordance with Frontiers’ policies.

This retraction was approved by the Chief Executive Editor of Frontiers. The authors received a communication regarding the retraction and had a chance to respond. This communication has been recorded by the publisher.

Frontiers would like to thank the users on PubPeer for bringing the published article to our attention.

